# Arrhythmias in Cardiac Amyloidosis

**DOI:** 10.19102/icrm.2018.090301

**Published:** 2018-03-15

**Authors:** Roy M. John

**Affiliations:** ^1^Department of Medicine, Cardiology Division, Vanderbilt University, Nashville, TN, USA

**Keywords:** Arrhythmias, atrial fibrillation, cardiac amyloidosis, heart block, ventricular arrhythmias

## Abstract

Arrhythmias are common in cardiac amyloidosis and vary based on the amyloidosis type. Conduction defects and atrial arrhythmias are more prevalent in transthyretin amyloidosis compared with light chain amyloidosis, and this difference might be a reflection of the longer survival time in the former. This review summarizes the available literature on arrhythmias in this increasingly recognized form of cardiomyopathy and raises the importance of performing systematic data collection to improve outcomes.

## Introduction

Amyloidosis is a collection of diseases characterized by extracellular deposition of the fibrillar proteinaceous material, amyloid, in tissues and organs. Amyloid can be formed from several precursor proteins that misfold into toxic oligomers, which then aggregate with proteoglycan and serum amyloid proteins to form amyloid infiltrates. The vast majority of cardiac amyloidosis (CA) is caused by one of two proteins: light chain or transthyretin (TTR). Light-chain (AL) amyloidosis is a hematological disorder of proliferation of an abnormal plasma cell clone that overproduces lambda or, less commonly, kappa light chains. The abnormal circulating light chains form amyloid that can affect multiple organs.^1^ AL amyloidosis has equal prevalence in both sexes and typically manifests between the ages of 40 and 80 years. Therapy is primarily aimed at managing plasma cell dyscrasia using chemotherapy or stem cell transplantation.

TTR is a pre-albumin produced by the liver and functions as a transporter of thyroxine and retinol in its normal tetrameric form. The monomeric form of TTR tends to misfold to produce amyloid fibrils that are deposited in tissues. Amyloidosis resulting from TTR is termed ATTR.^2^ There are two types of TTR-related amyloidosis. One is ATTR wild-type (ATTRwt), in which amyloid from normal TTR protein is deposited over a period of decades. ATTRwt has a strong male predilection, and patients typically present between the ages of 60 years and 95 years with carpal tunnel syndrome preceding cardiac involvement. Because of its late presentation, ATTRwt is also referred to as senile amyloidosis. The second type of ATTR is due to mutant TTR (ATTRm) causing familial amyloidosis; patients are generally born with a pathological mutation in the *TTR* gene, resulting in accelerated breakdown of TTR to amyloid. Over 80 different amyloidogenic missense point mutations have been described. Val-30-Met is the most common pathogenic mutation globally and is the leading cause of familial amyloid polyneuropathy. Another important mutation, Val-122-Ile, is seen in 3% to 4% of African Americans and is the most common mutation leading to familial CA. Patients with ATTRm present at an earlier age (40 to 75 years) than those with ATTRwt and demonstrate a slight male predominance. The clinical manifestation of ATTRm is driven by the specific mutation, with phenotypes ranging from exclusive neurological involvement to CA with overlapping patterns; however, most affected patients have some degree of multisystem involvement.^3^ Chemotherapy has no role in ATTR. Clinical trials of various therapeutic agents that modify or inhibit amyloid fibril formation are in progress. Prognosis for ATTR is generally better than that for AL amyloidosis.^4^ CA can also occur in secondary amyloidosis resulting from chronic inflammation, but this is rare, occurring in only 2% of cases **(Table 1)**.

**Figure 1:**
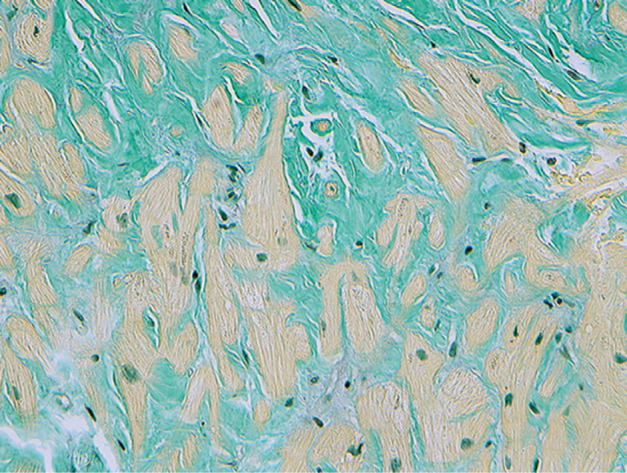
A high-powered view of a section of an endomyocardial biopsy in a patient with transthyretin amyloidosis stained with sulfated Alcian blue. The green staining is amyloid and the yellow staining is the myocardium. Note the extensive replacement of the myocardium by extracellular amyloid deposits.

The cardiac manifestations of amyloidosis include marked left ventricular (LV) thickening (due to amyloid fibril deposition) with electrocardiogram (ECG) voltage that is disproportionately low for the degree of LV thickening and associated diastolic (rather than systolic) heart failure. Clinical examination and echocardiography show restrictive cardiomyopathy features. Cardiac arrhythmias are common and are an important cause of morbidity and mortality. Diagnosis is based on imaging studies and biopsy. Cardiac magnetic resonance imaging (MRI) shows increased extracellular volume on T1 imaging, as well as patterns of late gadolinium enhancement in later stages.^5,6^ Bone-seeking radioisotopes have a high sensitivity for ATTR and are useful for the detection of subclinical CA when the MRI and ECG may still be unrevealing. ^99m^Tc-pyrophosphate for myocardial imaging is readily available and is currently the tracer of choice for the diagnosis of TTR CA. It is also useful for differentiation from AL CA as there is minimal or no myocardial uptake of the tracer in AL CA.^7^ Endomyocardial biopsy typically shows deposition of amyloid in the extracellular and perivascular spaces that can be specifically stained with sulfated Alcian blue **(Figure 1)**.

## Pathophysiology of arrhythmias in cardiac amyloidosis

Cardiac involvement is invariable in ATTRwt. In the familial TRR amyloidoses (ie, ATTRm), the prevalence of CA varies with the specific mutation. AL amyloidosis may have minimal or severe cardiac involvement, with 50% of patients showing CA. Regardless of the cause of amyloid production, CA is characterized by extracellular amyloid deposition throughout the heart, including the cardiac conduction tissue and valves. The ventricles are non-dilated and thickened, with restrictive filling. Elevated filling pressures result in atrial dilation despite atrial wall thickening from amyloid deposition. Atrial arrhythmias and loss of atrial mechanical function with atrial thrombus formation are important risk factors for cardioembolic stroke.^8,9^

The histology of CA is amyloid infiltration of the extracellular spaces separating and distorting the myocardial cells. Myocardial scarring and patchy fibrosis that are typical of chronic ischemic cardiomyopathy or other non-ischemic cardiomyopathies are not described in CA. Hence, the exact mechanism of arrhythmias in CA is less well defined and is likely to be multifactorial. Small vessel disease due to perivascular amyloid infiltration associated with impaired vasodilation is a likely substrate for myocardial ischemia, especially in AL.^10^ Patients with CA occasionally report angina-type chest pains. Persistent cardiac troponin elevation is a feature of AL CA and, to a lesser extent, of ATTR. This increase in troponin may represent myocardial ischemia from small vessel occlusion or direct toxic effects of AL light chains. AL amyloid has been demonstrated to impair myocyte function and calcium release by increasing oxidative stress.^11^ In a zebrafish model, injecting AL light chains isolated from AL cardiomyopathy patients impaired cardiac function and led to cell death.^12^ Thus AL amyloidosis is a toxic, infiltrative cardiomyopathy. Inflammatory cell damage and separation of myocytes by amyloid fibrils would explain conduction abnormalities, atrial arrhythmias, and loss of atrial contractility. In the ventricles, non-sustained ventricular tachycardia (VT) is the most common form of arrhythmia. Sustained monomorphic VT is uncommon, as most unexpected lethal ventricular arrhythmias are due to polymorphic VT or ventricular fibrillation (see later).

TTR amyloidosis, especially ATTRwt, has a protracted course with a median survival of 60 months following presentation with heart failure symptoms.^13^ Conversely, AL amyloidosis has a high mortality rate once cardiac involvement becomes apparent. Several adverse prognostic indicators have been described, including elevated N-terminal pro-brain natriuretic peptide (NT-pro-BNP) and troponin levels, diastolic dysfunction on echocardiography, and the extent of extracellular volume and late gadolinium enhancement on MRI.^14^ In general, arrhythmia interventions are not likely to be beneficial in cases of severe cardiac involvement, as myocardial dysfunction leads to electromechanical dissociation, the predominant terminal event in patients with CA.

There are few systematic studies of electrophysiological abnormalities and arrhythmias associated with CA and their appropriate management. Most available data are based on case reports or series from single centers. The nature, incidence, and management of arrhythmias differ based on the type of amyloidosis and extent of cardiac involvement.

## Conduction system disease and the role of cardiac pacing

The conduction system is affected in all forms of CA. Atrioventricular (AV) conduction delay or block is more common than sinus node disease: despite the high frequency of atrial involvement with amyloidosis, sinus node disease appears to be less common and relevant reports are mostly limited to isolated events in patients with ATTRm. In some reports, transient sinus node dysfunction occurred in association with autonomic dysfunction occurring spontaneously or during general anesthesia induction.^15^ An intracardiac electrophysiology (EP) evaluation of sinus node function in 25 patients with AL amyloidosis revealed normal sinus node function in 88% of those examined.^16^ Sayed et al. reported data from implanted loop recorders in 20 patients with Mayo stage III AL CA who were symptomatic with syncope or presyncope. Persistent sinus bradycardia with pauses requiring cardiac pacing was only detected in one patient.^17^

**Figure 2:**
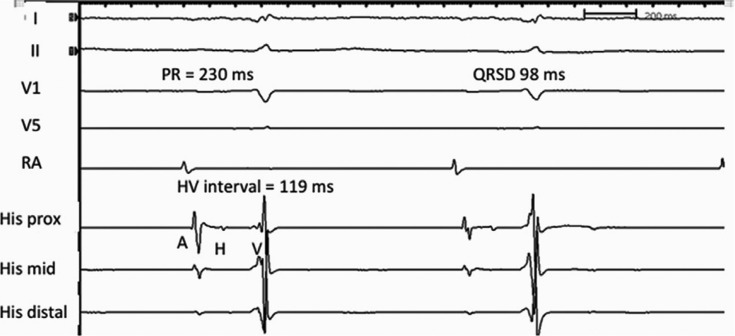
Intracardiac recording of a His bundle in a patient with ATTRwt cardiac amyloidosis and syncope. First-degree heart block is present with a PR interval of 230 ms. The PR prolongation is due to a conduction delay in the His-Purkinje system with an HV interval of 119 ms. Note the relatively narrow QTS duration of 98 ms despite marked prolongation of the HV interval. ATTRwt: wild type transthyretin amyloidosis; HV: His bundle–ventricular; QRSD: QRS duration; RA: right atrium.

**Figure 3:**
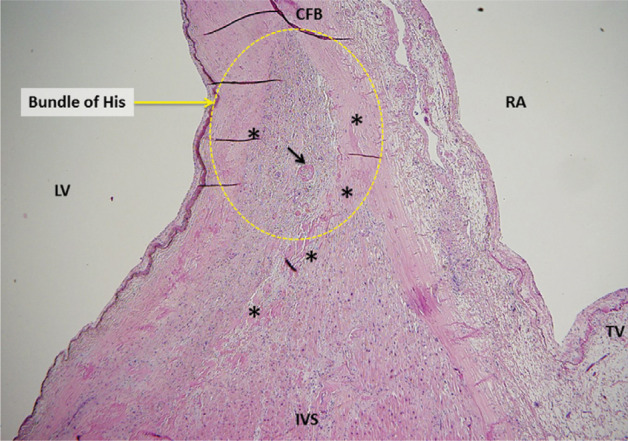
Hematoxylin and eosin staining of the region of the central fibrous body and His bundle in a patient who died of heart failure without a premortem diagnosis of cardiac amyloidosis. The patient had first-degree AV block but no higher grades of AV block. Autopsy showed extensive amyloid infiltration of the myocardium. The yellow circle represents the area of the His bundle. The pink amorphous eosin staining material marked by asterisks (*) represent amyloid deposits. The black arrow points to a small blood vessel with perivascular amyloid infiltration. CFB: central fibrous body; IVS: interventricular septum; LV: left ventricle; RA: right atrium; TV: tricuspid valve.

Conduction defects in the His-Purkinje system are more common and are associated with symptomatic AV block. First-degree AV block is often due to a delay in the His-Purkinje system level with preserved conduction at the nodal level. EP studies show an abnormal His bundle–ventricular (HV) interval in most patients with AL and ATTR CA.^16,18^ However, the incidence of symptomatic AV block is higher in ATTR, possibly because these patients are older and have better survival. In our series of 18 patients with advanced CA who underwent EP studies primarily for supraventricular arrhythmias, the mean (± standard deviation) HV interval was 87 ± 27 ms, despite a relatively narrow QRS duration (119 ± 32 ms).^18^ Prolongation of the HV interval with preserved QRS duration is well recognized in CA **(Figures 2** and **3)** and may represent diffuse amyloid infiltration of the bundles, creating equal delays in both the right and left branches, yielding a disproportionately narrow QRS.^16^

Approximately 25% to 35% of ATTRm and 45% of ATTRwt patients receive pacemaker implants. While cardiac pacing provides symptomatic relief, it does not change the overall prognosis.^19^ Based on the known pathophysiology of CA and the high incidence of conduction abnormalities, the threshold for pacemaker implantation should be low to prevent recurrent syncope. Prophylactic pacemaker implants have been suggested for all patients with ATTR CA. However, the downside of transvenous pacemakers is the possibility of worsening tricuspid regurgitation due to endocardial leads that cross the valve, impinging on thickened tricuspid leaflets. The advent of leadless pacemakers might partially mitigate this problem.

When the predominant rhythm is sinus, consideration should be given to atrial synchronized ventricular pacing, as maintenance of AV synchrony with atrial synchronized ventricular pacing may be important for maintaining cardiac output in the setting of diastolic dysfunction and preload dependence. A combination of peripheral vasoconstrictors and atrial pacing can be helpful in maintaining an adequate blood pressure in patients with severe autonomic neuropathy and chronotropic incompetence. When ventricular pacing is necessary, there is concern that dysynchrony from right ventricular (RV)-only pacing may cause further deterioration of ventricular function. Observations during echocardiography suggest improved cardiac output from biventricular pacing (personal communication). However, there are currently no formal studies to support this premise.

## Atrial arrhythmias

Atrial fibrillation (AF) or atrial tachycardia (AT) is common in CA, especially as the disease progresses. In a group of patients from Sweden with familial amyloid polyneuropathy, 20% had previously undetected atrial arrhythmias suggesting possible amyloid atriopathy, even in the absence of overt CA.^20^ Arrhythmia is more common in ATTRwt due to the older age at presentation and the higher prevalence of age-related AF. In one series, 62% of patients with ATTRwt CA had AF.^20^ AF or AT is often highly symptomatic and poorly tolerated, mostly due to rapid ventricular rates and an irregular ventricular response that impairs ventricular filling and contractility. Given that atrial contractility is often diminished in CA, the loss of atrial contribution is less likely to be the mechanism for deterioration. Ventricular rate control can be difficult since β-blockers and calcium blockers are not well tolerated due to hypotension and their negative inotropic effects. Although digoxin is known to bind amyloid tissue and increase the risk of toxicity, its use in low doses is usually tolerated, although its role in rate control is limited. Amiodarone provides a good option for AF control and is fairly well tolerated when administered orally. Alternative drugs such as dofetilide can be useful for maintaining sinus rhythm when used cautiously with close monitoring of the QT interval. Anticoagulation is usually well tolerated and should be used for all atrial arrhythmias. If there is evidence for non-contractility of the atria, then there is a case to be made for anticoagulation even in sinus rhythm, as thromboembolism may occur with stasis of blood in the left atrium and appendage.^21^

**Figure 4:**
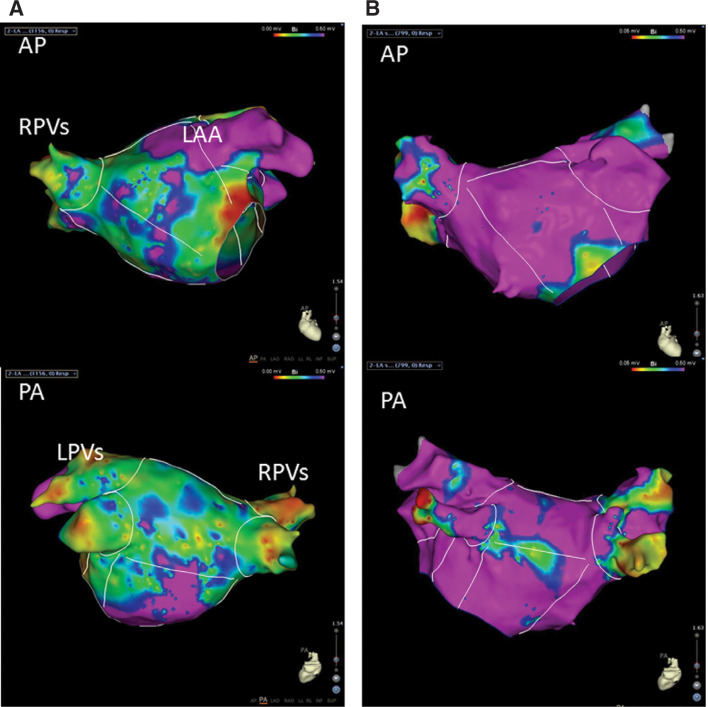
LA voltage map comparison. **A:** LA voltage map of a patient with AL amyloidosis and atrial tachycardia. **B:** LA voltage map of an age-matched patient with persistent AF but without amyloidosis. The purple area represents bipolar voltage greater than 0.5 mV, and blue, green, and yellow represent gradients of voltage between 0.5 and 0.05 mV. The red areas represent voltage below 0.05 mV. There are far more extensive low-voltage areas in the LA of the patient with AL amyloidosis compared with the control patient. While this finding of low voltage in the LA is not specific to any disease process, an extensive voltage abnormality with relatively normal LA dimensions should raise suspicion for cardiac amyloidosis.^18^ AL: light chain; AF: atrial fibrillation; AP: anteroposterior; LA: left atrial/left atrium; LAA: left atrial appendage; RPVs: right pulmonary veins; PA: posteroanterior; LPVs: left pulmonary veins.

AF in amyloidosis tends to have longer cycle lengths and may appear organized.^18^ This is likely due to amyloid deposition separating out the atrial myocyte bundles and creating a marked delay in intra-atrial conduction. In our series, left atrial voltage mapping revealed significantly lower voltages in amyloid patients compared with an age-matched patient population with persistent AF **(Figure 4)**.^18^ However, ablation attempts to correct what is often interpreted as left atrial tachycardia on surface ECG often yield only limited success. Despite acute termination and restoration of sinus rhythm with ablation, the recurrence rate is high. In our series, the recurrence rate at one year was 83% for CA patients compared with 25% in the non-CA, persistent AF patient group.^18^ In addition, procedural complications are higher in CA patients with poor tolerance of fluid shifts and general anesthesia. Acute chronic renal failure and pulmonary congestion are common in the postprocedural period and may require lengthy hospitalization for correction.

When the heart rate is difficult to control, AV nodal ablation with ventricular pacing has the benefits of rate control and rate regularization that improve cardiac output and offer symptomatic improvement.^22^ For ventricular pacing following AV nodal ablation, our practice is to aim for biventricular pacing or placement of the RV lead in the high septal or para-His regions to minimize further depression of ventricular function due to ventricular dysynchrony from RV pacing.

Isolated atrial amyloidosis due to atrial natriuretic peptide depositing as amyloid fibrils is seen in up to one-third of patients with persistent AF undergoing valve surgery and is unrelated to the atrial arrhythmias of generalized CA. Isolated atrial amyloidosis is a disease of the elderly, with a female preponderance.^23^

## Syncope

Postural hypotension resulting from autonomic dysfunction is a common finding in AL amyloidosis and a cause of syncope in this patient population. Diuretics tend to aggravate symptoms. Midodrine, an alpha 1 adrenergic stimulant, is often necessary in high doses to maintain blood pressure. The supine hypertension that is common with midodrine use is not seen in CA, so large doses can be employed without concern. Cardiac arrhythmias such as heart block and ventricular arrhythmias are more important causes of syncope and may be a harbinger of sudden death, but careful history taking and examination can often differentiate one from the other. Ambulatory monitoring and EP studies can be helpful, especially for the detection of infra-Hisian AV block. Atrial arrhythmias with rapid ventricular rates can cause a precipitous drop in cardiac output, resulting in syncope. In advanced CA, another mechanism for syncope is vasodilation with an inability to increase cardiac output due to poor contractility reserve. Patients may present with exertional syncope. Prophylactic midodrine prior to planned exercise can be helpful.

## Ventricular arrhythmias

As end-stage heart failure is the major driver of fatality in CA, managing ventricular arrhythmias has assumed a lesser role in overall outcome. AL amyloid, with its more precipitous downward course after heart failure onset, has a higher incidence of ventricular arrhythmia compared with that of ATTR disease. However, there are few systematic studies on the prevalence of ventricular arrhythmias in CA available to date. In a study of 195 patients with AL amyloidosis, 24-hour Holter recordings revealed non-sustained VT in 27% of patients with advanced AL CA.^24^ In this study, mortality was high (88%), suggesting a patient population in a late stage of the disease. The incidence of sudden death was no different between those with and without non-sustained VT, implying little prognostic importance regarding the finding of non-sustained VT alone. In a more recent study with implanted loop monitors in 20 patients with AL CA, non-sustained VT was observed in only one transmission.^17^ In both studies, recorded sudden deaths were mostly associated with terminal bradycardia followed shortly by pulseless electrical activity.

Monomorphic VT is occasionally inducible during EP study or is noted in patients with an implantable cardioverter-defibrillator (ICD), but it is an infrequent event in CA compared with other cardiomyopathies.^18^ Ventricular fibrillation storm provoked by monomorphic premature ventricular complexes of Purkinje origin was reported in two patients with possible AL amyloidosis, supporting the hypothesis of an inflammatory response to AL amyloid.^25^ Catheter ablation targeting the Purkinje fibers to trigger fibrillation is helpful in acute arrhythmia control.

## Sudden death and the role of implantable cardioverter-defibrillators

The most frequent documented terminal event in CA is pulseless electrical activity or agonal bradycardia.^24^ Even in sudden death, the mechanism is commonly electromechanical dissociation from end-stage heart failure. As a result, there has been very little enthusiasm for the ICD in patients with CA. Arrhythmic sudden death is far more common in AL cardiomyopathy. However, the prognosis of AL amyloidosis with heart failure has historically been dismal, with survival lasting less than 12 months after diagnosis, representing a relative contraindication to ICD implantation. When life expectancy is greater than one year, secondary prevention ICD is a reasonable consideration, following a discussion of the risks and benefits. There is a tendency for a higher defibrillation threshold in patients with CA, but first shock efficacy is generally comparable to that reported in the SCD-Heart Failure Trial study.^26^ Primary prevention ICD implantation in CA patients is associated with a high rate of appropriate discharges for ventricular arrhythmias in AL patients (32% in the first year, in one series) but does not translate to improved survivial.^27,28^ With improved treatment options for AL amyloidosis, it is time to revisit the issue of primary prevention ICDs. However, the prediction of risk for arrhythmic sudden death does not follow the available guidelines for other forms of heart disease. An ICD would be a reasonable consideration for unexplained syncope or recurrent episodes of non-sustained VT in the early stages of AL amyloidosis, when the levels of biomarkers such as troponin and NT-pro-BNP are low. Primary prevention ICD may also have a role in patients being considered for cardiac transplantation.

## Future directions and conclusions

A combined multicenter effort to define the cardiac arrhythmias associated with CA is essential to gauge the survival benefits from early antiarrhythmic prophylaxis in CA. The formation of the Amyloid Research Consortium is an encouraging move in this direction (http://www.arci.org). Early pacemaker implantation in patients at high risk for heart block may also serve as arrhythmia monitoring with respect to data gathering. With the high rate of pacemaker implantation in ATTR amyloidosis, a trial of biventricular pacing in patients with heart block is feasible and important to clarify the role of ventricular synchrony in this unique form of cardiomyopathy. In the more malignant AL amyloidosis, the role of ICDs has thus far been assessed largely in the later stages, when severe heart failure negates any benefit from defibrillation. ICD should be evaluated in the earlier stages of AL CA, when arrhythmic sudden death is likely to be a greater risk.

There has been considerable progress in our understanding of amyloidosis pathophysiology and treatment in recent years. Drugs aimed at preventing new amyloid formation achieve very favorable responses in some patients, and formal trial results are awaited. Early diagnosis and management by experts are key to improving outcomes in CA. The disease can no longer be dismissed as a terminal illness with limited therapeutic options.

## Figures and Tables

**Table 1:** Types and Features of CA

Amyloidosis Nomenclature	Precursor Protein	Clinical Features		Cardiac Involvement		Laboratory Findings		Treatment	
AL	Light chains	•	Age: 40+ years	•	CA present in 50%	•	Elevated free lambda or kappa light chains	•	Chemotherapy
		•	Males = females	•	Heart block			•	Stem cell transplantation
		•	Multiorgan involvement with autonomic dysfunction, periorbital edema	•	Atrial arrhythmias				
				•	Ventricular arrhythmias				
ATTRwt	Wild-type (normal) TTR	•	Age: 60+ years	•	CA present in 100%	•	Positive ^99m^TcPyP or ^99m^Tc-DPD scan	•	Supportive therapy for heart failure
		•	Males > females	•	Heart block			•	Pacemaker implant for AV block
		•	Carpal tunnel syndrome	•	Atrial fibrillation			•	Investigational drugs that prevent TTR amyloidogenesis
				•	VT less common				
ATTRm	Mutant TTR	•	Age of onset variable: 40+ years in Val-30-Met and 60+ years in Val-122-Ile mutations	•	Cardiac involvement is mutation dependent	•	Positive ^99m^Tc scan	•	Genetic testing defines the TTR mutation
		•	Males = females	•	100% in Val-122-Ile mutation				
		•	Polyneuropathy is common						
AA	Serum amyloid A (an acute phase protein)	•	Occurs in those older than 20 years of age, in association with severe chronic inflammatory disease	•	Cardiac involvement is rare (2%)	•	Markers of inflammation		
		•	Hepatic and splenic enlargement			•	Proteinuria		
IAA	Atrial natriuretic peptide	•	Occurs in association with chronic atrial fibrillation in older females but also seen with chronic heart disease	•	Amyloid deposition in atria, left more than right	•	Diagnosis made from surgical specimens and autopsy
						•	Unclear clinical importance		

CA: cardiac amyloidosis; AL: light chain amyloidosis; ATTRwt: wild-type transthyretin amyloidosis; TTR: transthyretin; VT: ventricular tachycardia; ^99m^Tc-PyP: technetium pyrophosphate; ^99m^Tc-DPD: technetium 3,3-diphosphono-1,2-propanodicarboxylic acid; AV: atrioventricular; ATTRm: mutant transthyretin amyloidosis; AA: secondary amyloidosis; IAA: isolated atrial amyloidosis. Adapted from Falk et al.^1^

## References

[1] Falk RH, Alexander KM, Liao R, Dorbala S (2016). AL (light chain) cardiac amyloidosis: a review of diagnosis and therapy. J Am Coll Cardiol..

[2] Gertz MA, Benson MD, Dyck PJ (2015). Diagnosis, prognosis, and therapy of transthyretin amyloidosis. J Am Coll Cardiol..

[3] Coelho T, Maurer MS, Suhr OB (2013). THAOS—The Transthyretin Amyloidosis Outcomes Survey: initial report on clinical manifestations in patients with hereditary and wild-type transthyretin amyloidosis. Curr Med Res Opin..

[4] Rapezzi C, Merlini G, Quarta CC (2009). Systemic cardiac amyloidosis: disease profiles and clinic course of the 3 main types. Circulation..

[5] Vogelsberg H, Mahrholdt H, Deluigi CC (2008). Cardiovascular magnetic resonance in clinically suspected cardiac amyloidosis: noninvasive imaging compared to endomyocardial biopsy. J Am Coll Cardiol..

[6] Banypersaud SM, Fontana M, Maestrini V (2015). T1 mapping and survival in systemic light-chain amyloidosis. Eur Heart J..

[7] Bokhari S, Castano A, Pozniakoff T, Deslisle S, Latif F, Maurer MS (2013). (99m)Tc-pyrophosphate scintigraphy for differentiating light-chain cardiac amyloidosis from the transthyretin-related familial and senile cardiac amyloidosis. Circ Cardiovasc Imaging..

[8] Nagakawa M, Tojo K, Sekijima Y, Yamazaki KH, Ikeda S (2012). Arterial thromboembolism in senile systemic amyloidosis: report of two cases. Amyloid..

[9] Feng D, Syed IS, Martinez M (2009). Intracardiac thrombosis and anticoagulation therapy in cardiac amyloidosis. Circulation..

[10] Dorbala S, Vangala D, Bruyere J (2014). Coronary microvascular dysfunction is related to abnormalities in myocardial structure and function in cardiac amyloidosis. JACC Heart Fail..

[11] Brenner DA, Jain M, Pimentel DR (2004). Human amyloidogenic light chains directly impair cardiomyocyte function through an increase in cellular oxidant stress. Circ Res..

[12] Mishra S, Guan J, Plovie E (2013). Human amyloidogenic light chain proteins result in cardiac dysfunction, cell death and early mortality in zebrafish. Am J Physiol Heart Circ Physiol..

[13] Pinney JH, Whelan CJ, Petrie A (2013). Senile systemic amyloidosis: clinical features at presentation and outcome. J Am Heart Assoc..

[14] Kumar S, Dispenzieri A, Lacy MQ (2012). Revised prognostic staging system for light chain amyloidosis incorporating cardiac biomarkers and serum free light chain measurements. J Clin Oncol..

[15] Chastan N, Baert-Desurmont A, Saugier-Veber P (2006). Cardiac conduction alterations in a French family with amyloidosis of the Finnish type with p.Asp187Tyr mutation in the GSN gene. Muscle Nerve..

[16] Reisinger J, Dubrey SW, Lavalley M, Skinner M, Falk RH (1997). Electrophysiologic abnormalities in AL (primary) amyloidosis with cardiac involvement. J Am Coll Cardiol..

[17] Sayed RH, Rogers D, Khan F (2015). A study of implanted cardiac rhythm recorders in advanced cardiac AL amyloidosis. Eur Heart J..

[18] Barbhaiya CR, Kumar S, Baldinger SH (2016). Electrophysiological assessment of conduction abnormalities and atrial arrhythmias associated with amyloid cardiomyopathy. Heart Rhythm..

[19] Givens RC, Russo C, Green P, Maurer MS (2013). Comparison of cardiac amyloidosis due to wild-type and V122l transthyretin in older adults referred to an academic medical center. Aging Health..

[20] Hornsten R, Pennlert J, Wilund U, Lindqvist P, Jensen SM, Suhr OB (2010). Heart complications in familial transthyretin amyloidosis: impact of age and gender. Amyloid..

[21] Nochioka K, Quarta CC, Claggett B (2017). Left atrial structure and function in cardiac amyloidosis. Eur Heart J Cardiovasc Imaging.

[22] Tan NY, Mohsin Y, Hodge DO (2016). Catheter ablation for atrial arrhythmias in patients with cardiac amyloidosis. J Cardiovasc Electrophysiol..

[23] Rocken C, Peters B, Juenemann G (2002). Atrial amyloidosis: an arrhythmogenic substrate for persistent atrial fibrillation. Circulation..

[24] Dubrey SW, Cha K, Anderson J (1998). The clinical features of immunoglobulin light-chain (AL) amyloidosis with heart involvement. Q J Med..

[25] Mlcochova H, Saliba WI, Burkhardt DJ (2006). Catheter ablation of ventricular fibrillation storm in patients with infiltrative amyloidosis of the heart. J Cardiovasc Electrophysiol..

[26] Varr BC, Zarafshar S, Coakley T (2014). Implantable cardioverter-defibrillator placement in patients with cardiac amyloidosis. Heart Rhythm..

[27] Kristen AV, Dengler TJ, Hagenbart U (2008). Prophylactic implantation of cardioverter-defibrillator in patients with severe cardiac amyloidosis and high risk for sudden cardiac death. Heart Rhythm..

[28] Lin G, Dispenzieri A, Kyle R, Grogan M, Brady PA (2013). Implantable cardioverter defibrillators in patients with cardiac amyloidosis. J Cardiovasc Electrophysiol..

